# Supplementary material for: Clonally expanded B cell populations are a distinguishing feature of chronic Chagas cardiomyopathy
Source: medRxiv. 2026 Oct 30:2026.10.28.26366745. Preprint. [Version 1] doi: 10.64898/2026.10.28.26366745 (PMC13734189; doi:10.64898/2026.10.28.26366745)
Supplement: Supplementary file 1 [file NIHPP2026.10.28.26366745v1-supplement-25.pdf]

medRxiv preprint doi: https://doi.org/10.64898/2026.10.28.26366745; this version posted October 30, 2026. The copyright holder for this preprint (which was not certified by peer review) is the author/funder, who has granted medRxiv a license to display the preprint in perpetuity.
It is made available under a CC-BY 4.0 International license .

Supplemental Table 1: Demographics of nested case-controls for early CCC progression. P value calculated by Fisher's exact test for categorical variables and two-sided t-test for continuous variables.

	Females			Males		
	**Stable indeterminate (A → A)**	**Early progressors (A → B1/B2)**	**P value**	**Stable indeterminate (A → A)**	**Early progressors (A → B1/B2)**	**P value**
**Number**	18	9		18	9	
**Age (median [IQR])**	51.50 [46.00, 55.75]	50.00 [48.00, 56.00]	0.979	42.50 [38.25, 52.75]	45.00 [37.00, 51.00]	0.898
**Time to progression (median [IQR])**	NA	2.40 [1.67, 3.09]	NA	NA	1.83 [1.50, 2.40]	NA
**Storage Media**						
RNA later(%)	3 (16.7)	1 (11.1)	1	5 (27.8)	4 (44.4)	0.423
DNA/RNA Shield (%)	15 (83.3)	8 (88.9)		13 (72.2)	5 (55.6)	
**Hypertension (%)**	9 (50.0)	6 (66.7)	0.683	11 (61.1)	5 (55.6)	1
**Diabetes(%)**	2 (11.1)	0 (0.0)	0.538	1 (5.6)	0 (0.0)	1
**BMI (median [IQR])**	28.12 [26.31, 31.82]	30.82 [28.08, 36.48]	0.258	30.19 [26.74, 35.33]	28.30 [26.91, 30.45]	0.607

Supplemental Table 2: Demographics of nested case-controls for advanced CCC progression. P value calculated by Fisher's exact test for categorical variables and two-sided t-test for continuous variables.

	Females			Males		
	**Stable early CCC (B1 → B1)**	**Advanced progressors (B1 → B2/C)**	**P value**	**Stable early CCC (B1 → B1)**	**Advanced progressors (B1 → B2/C)**	**P value**
**Number**	20	10		22	14	
**Age (mean(sd))**	57.70 (11.11)	63.80 (14.64)	0.213	60.64 (9.40)	63.79 (11.09)	0.367
**Time to progression (median [IQR])**	NA	1.98 [1.49, 2.59]	NA	NA	2.03 [1.30, 3.85]	NA
**Storage Media**						
RNA later (%)	8 (40.0)	7 (70.0)	0.245	9 (40.9)	5 (35.7)	1
DNA/RNA Shield (%)	12 (60.0)	3 (30.0)		13 (59.1)	9 (64.3)	
**Hypertension (%)**	14 (70.0)	8 (80.0)	0.682	14 (63.6)	10 (71.4)	0.727
**Diabetes(%)**	3 (15.0)	0 (0.0)	0.532	5 (22.7)	2 (14.3)	0.681
**BMI (median [IQR])**	27.55 [26.01, 32.14]	26.95 [23.29, 30.56]	0.333	28.67 [27.04, 33.28]	31.25 [25.42, 33.73]	0.711

medRxiv preprint doi: https://doi.org/10.64898/2026.10.28.26366745; this version posted October 30, 2026. The copyright holder for this preprint (which was not certified by peer review) is the author/funder, who has granted medRxiv a license to display the preprint in perpetuity.
It is made available under a CC-BY 4.0 International license .

Supplemental Table 3-14: see corresponding excel sheet

Supplemental Table 15: Demographics of cross-sectional samples used for single-cell RNA sequencing

	**Non-Chagas**		**Chagas**			**pvalue**
stage	**A Neg**	**B1 Neg**	**A Pos**	**B Pos**	**C Pos**	
n	3	7	8	7	4	
Female (%)	3 (100.0)	7 (100.0)	8 (100.0)	7 (100.0)	4 (100.0)	1
Age (mean (SD))	56.67 (2.31)	63.57 (8.14)	54.62 (10.08)	61.57 (9.81)	61.50 (9.11)	0.354
Comorbidities						
Hypertension (%)	2 (66.7)	7 (100.0)	1 (12.5)	2 (28.6)	1 (25.0)	**0.003**
Diabetes(%)	0 (0.0)	0 (0.0)	1 (12.5)	0 (0.0)	0 (0.0)	1
BMI (mean (SD))	31.74 (8.49)	33.36 (6.94)	28.82 (5.29)	29.49 (4.02)	32.11 (5.89)	0.591
Parasite qPCR						
Positive	0(0)	0(0)	1 (12.5)	1 (14.3)	1 (25.0)	0.794
Indeterminate	0(0)	2 (28.6)	1 (12.5)	0(0)	0(0)	
Negative	3 (100.0)	4 (57.1)	6 (75.0)	6 (85.7)	3 (75.0)	
Missing	0(0)	1 (14.3)	0(0)	0(0)	0(0)	

Supplemental Table 17: Demographics of cardiac explant samples used for spatial transcriptomics

	**Non-Chagas Dilated Cardiomyopathy (NC-DCM)**	**Chronic Chagas Cardiomyopathy (CCC)**	**P value**
n	4	4	
Female (%)	2 (50.0)	2 (50.0)	1
Age (mean (SD))	51.50 (16.13)	54.50 (9.40)	0.759
LVEF % (median [IQR])	13.50 [11.50, 15.62]	15.00 [14.50, 23.50]	0.372
Heart failure etiology			
Valvular disease (%)	1 (25.0)	0 (0.0)	1
cardiotoxicity (%)	1 (25.0)	0 (0.0)	1
Idiopathic dilated cardiomyopathy (%)	2 (50.0)	0 (0.0)	0.429
Chagas cardiomyopathy (%)	0 (0.0)	4 (100.0)	**0.029**
Comorbidities			
Hypertension(%)	1 (25.0)	2 (50.0)	1.000
Diabetes(%)	0 (0)	2 (50.0)	0.429
BMI (median [IQR])	22.95 [22.40, 23.38]	26.40 [22.80, 29.50]	0.386

medRxiv preprint doi: https://doi.org/10.64898/2026.10.28.26366745; this version posted October 30, 2026. The copyright holder for this preprint (which was not certified by peer review) is the author/funder, who has granted medRxiv a license to display the preprint in perpetuity.
It is made available under a CC-BY 4.0 International license .

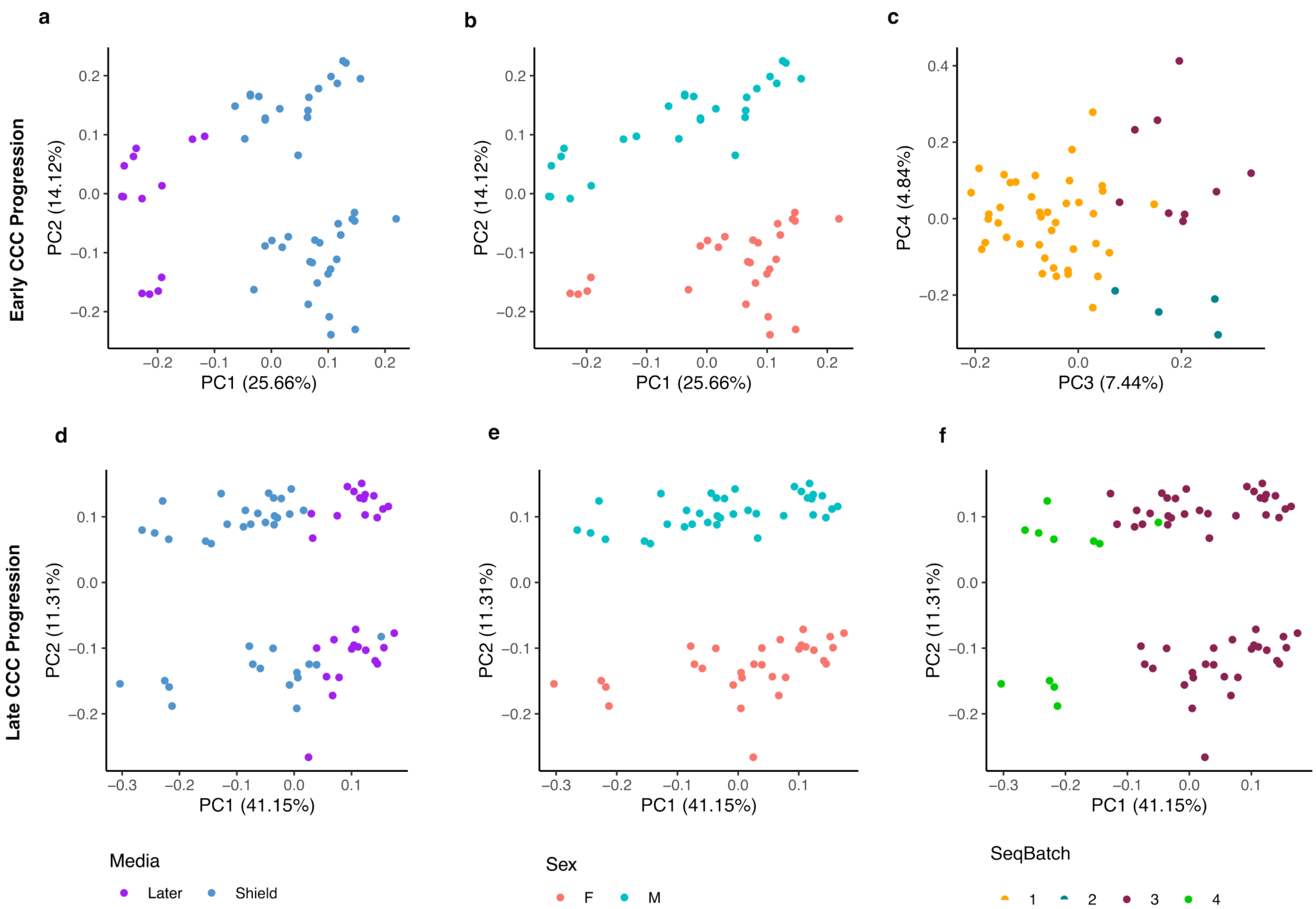

Supplementary Figure 1. Sequencing batch, storage media and sex are major drivers of variability in the bulk RNAseq data. PCA plots of bulk RNA sequencing samples in the early progression **(a-c)** and advanced progression **(d-f)** comparisons. Samples are colored by media **(a,d),** sex **(b, e)** or sequencing batch **(c,f).** Axis labels indicate the principal component and the percentage of variability that they explain in parentheses.

medRxiv preprint doi: https://doi.org/10.64898/2026.10.28.26366745; this version posted October 30, 2026. The copyright holder for this preprint (which was not certified by peer review) is the author/funder, who has granted medRxiv a license to display the preprint in perpetuity.
It is made available under a CC-BY 4.0 International license .

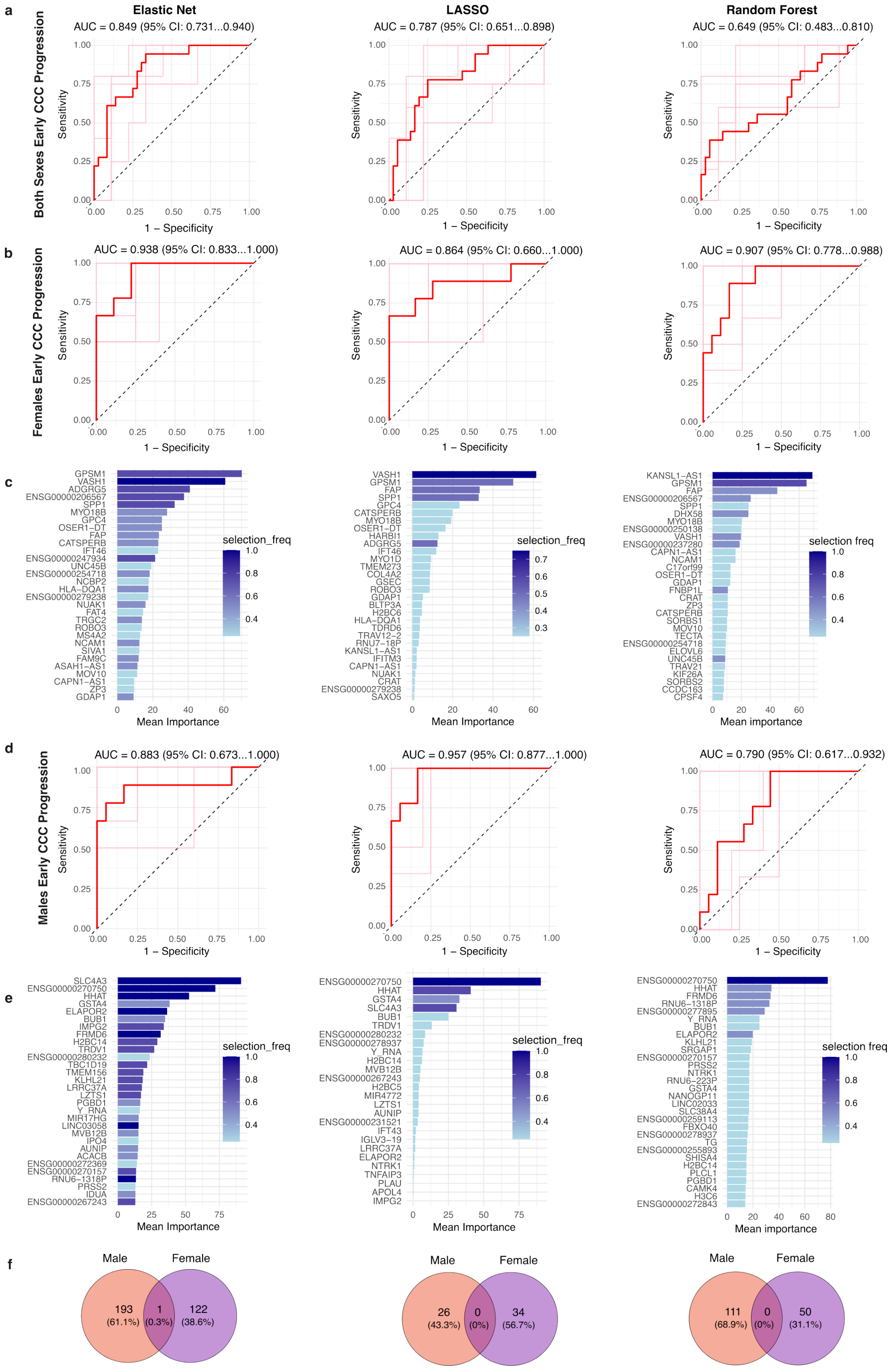

Supplementary Figure 2. Sex-specific elastic net models generally outperform other models of early CCC progression. a) ROC curve for elastic net, lasso and random forest models of early CCC progression using both sexes. The red line ROC curve indicates the overall result. The pink line indicates the results for each cross-validation fold. **b)** ROC curves for female-only models. **c)** Top 30 most important features for female-only models colored by selection frequency during nested cross-validation. **d)** ROC curves for

medRxiv preprint doi: https://doi.org/10.64898/2026.10.28.26366745; this version posted October 30, 2026. The copyright holder for this preprint (which was not certified by peer review) is the author/funder, who has granted medRxiv a license to display the preprint in perpetuity.
It is made available under a CC-BY 4.0 International license .

male-only models. **e)** Top 30 most important features for male-only model. **f)** Venn diagram of selected features for male-only and female-only models. For all figures, the (left) panel is for the elastic net models, (mid) for LASSO, and (right) for the random forest models.

medRxiv preprint doi: https://doi.org/10.64898/2026.10.28.26366745; this version posted October 30, 2026. The copyright holder for this preprint (which was not certified by peer review) is the author/funder, who has granted medRxiv a license to display the preprint in perpetuity.
It is made available under a CC-BY 4.0 International license .

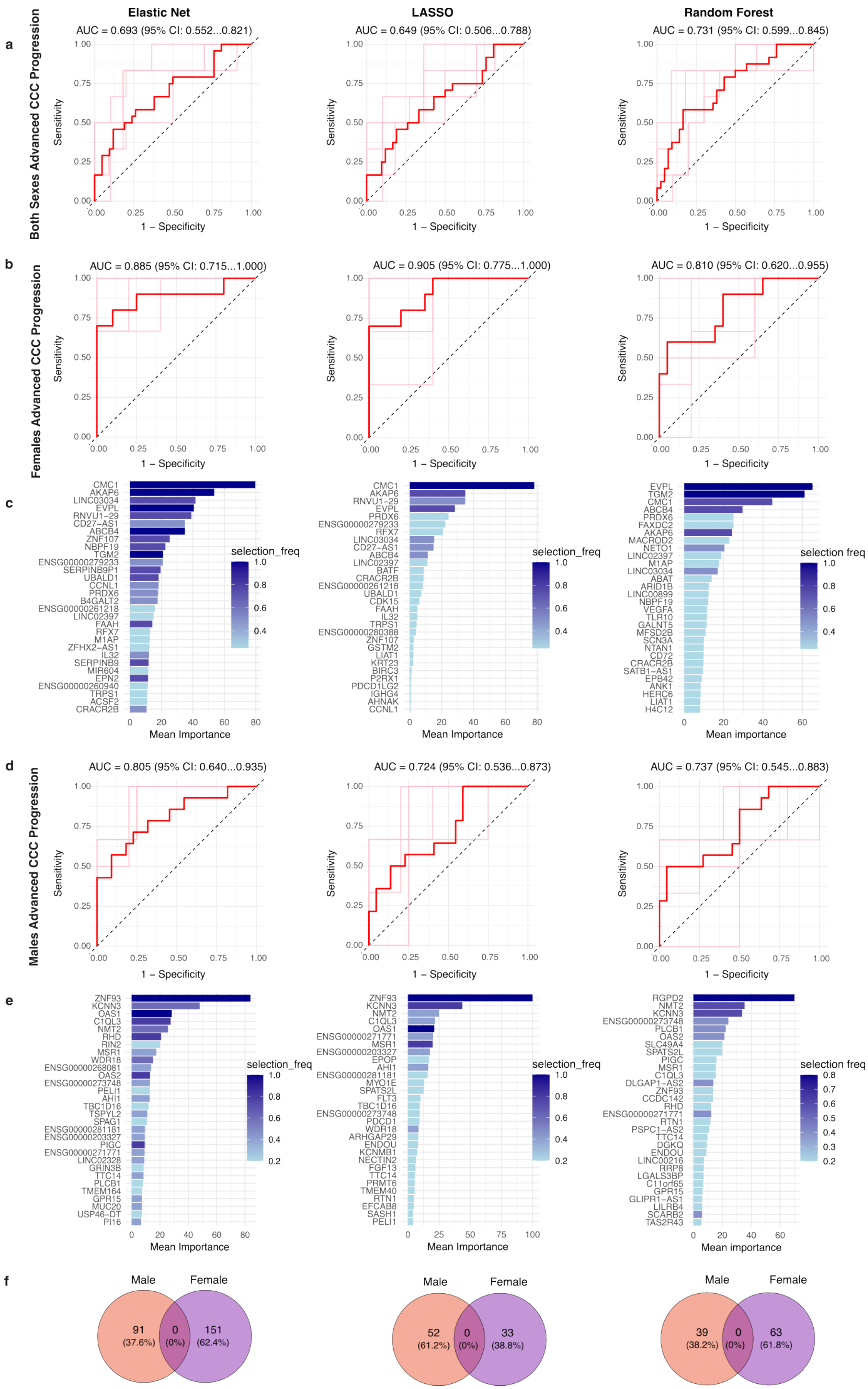

medRxiv preprint doi: https://doi.org/10.64898/2026.10.28.26366745; this version posted October 30, 2026. The copyright holder for this preprint (which was not certified by peer review) is the author/funder, who has granted medRxiv a license to display the preprint in perpetuity.
It is made available under a CC-BY 4.0 International license .

Supplementary Figure 3. Sex-specific elastic net models generally outperform other models of advanced CCC progression. a) ROC curve for elastic net, lasso and random forest models of advanced CCC progression using both sexes. The red line ROC curve indicates the overall result. The pink line indicates the results for each cross-validation fold. **b)** ROC curves for female-only models. **c)** Top 30 most important features for female-only models colored by selection frequency during nested cross-validation. **d)** ROC curves for male-only models. **e)** Top 30 most important features for male-only model. **f)** Venn diagram of selected features for male-only and female-only models. For all figures, the (left) panel is for the elastic net models, (mid) for LASSO, and (right) for the random forest models.

medRxiv preprint doi: https://doi.org/10.64898/2026.10.28.26366745; this version posted October 30, 2026. The copyright holder for this preprint (which was not certified by peer review) is the author/funder, who has granted medRxiv a license to display the preprint in perpetuity.
It is made available under a CC-BY 4.0 International license .

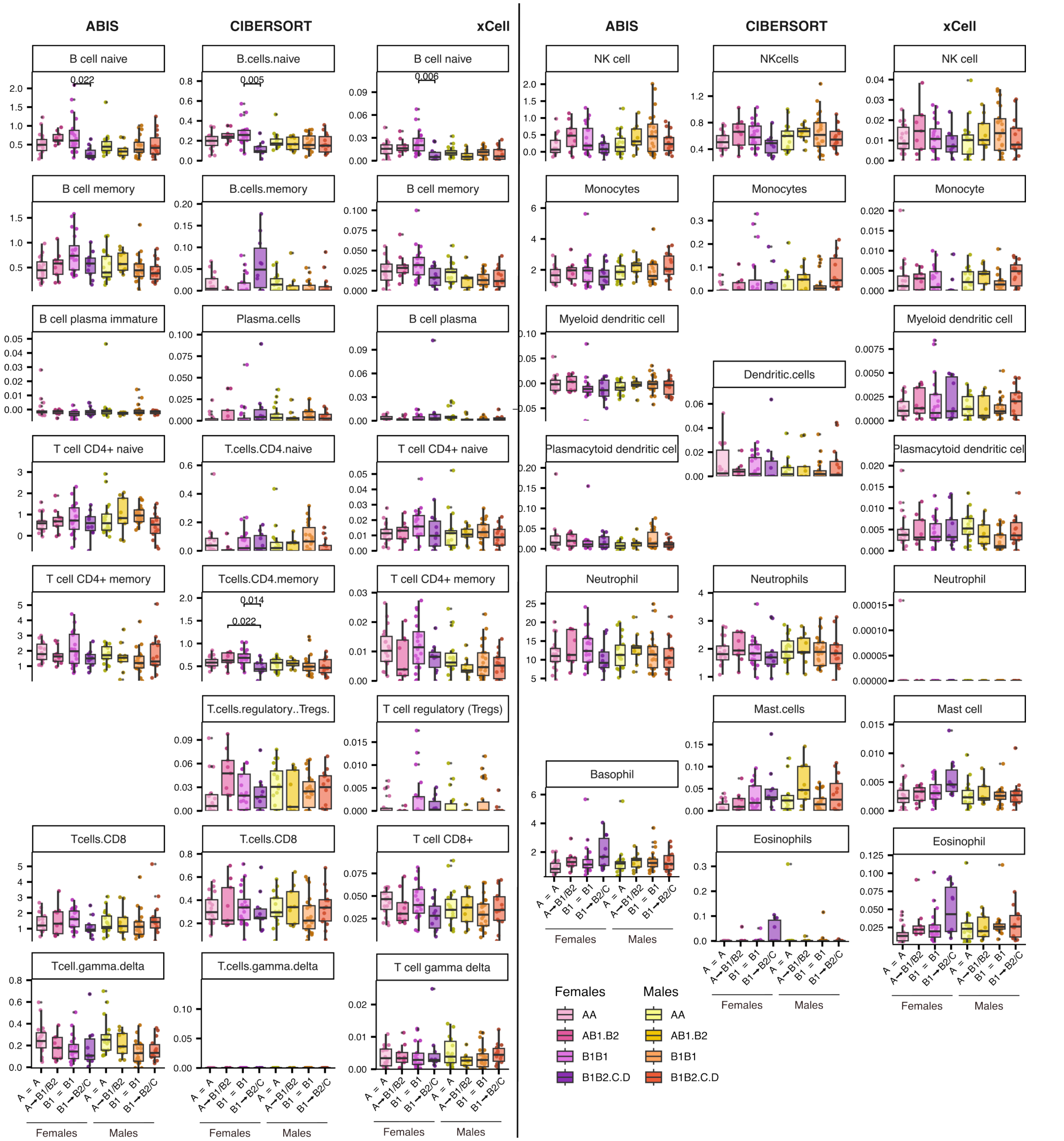

Supplementary figure 4. Multiple deconvolution tools consistently identify lower naïve B cell estimates in female advanced progressors. Immune cell deconvolution score (arbitrary values) by progression group stratified by sex and faceted by immune cell type and deconvolution algorithm. Statistics by linear regression (adjusting for sequencing batch and media), followed by estimated marginal means pairwise comparisons with FDR correction.

medRxiv preprint doi: https://doi.org/10.64898/2026.10.28.26366745; this version posted October 30, 2026. The copyright holder for this preprint (which was not certified by peer review) is the author/funder, who has granted medRxiv a license to display the preprint in perpetuity.
It is made available under a CC-BY 4.0 International license .

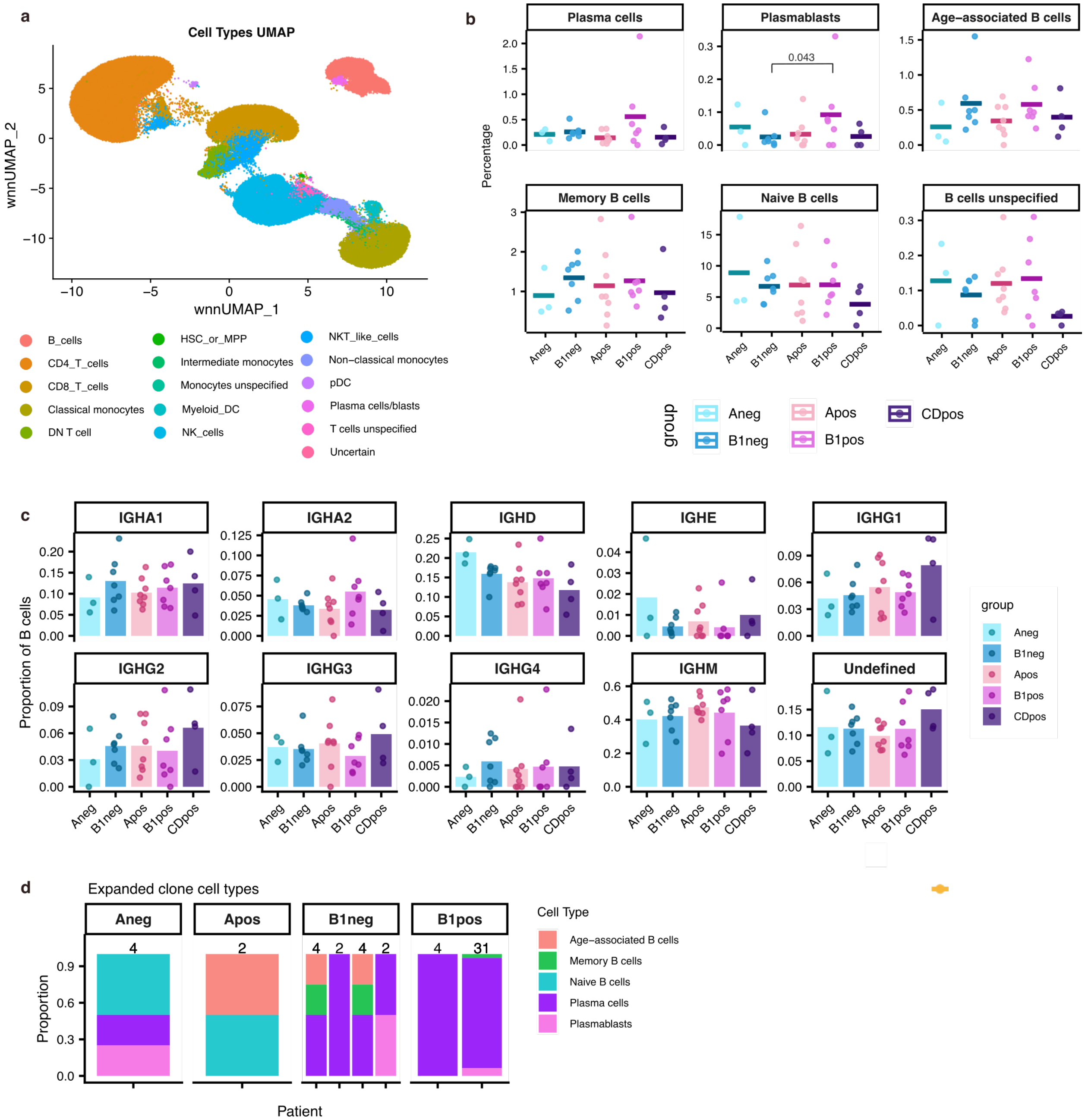

Supplementary figure 5. Single-cell RNAseq also shows evidence of B cell clonal expansion and class-switching. a) UMAP showing all cell type annotations. **b)** B cell subtype percentage by disease (pos = Chagas, neg = non-Chagas) and stage. Statistics by beta regression, followed by estimated marginal means pairwise comparisons with FDR correction. **c)** Isotype proportions for B cells by stage and disease. Bar indicates mean value. **d)** B cell subtype of expanded clones by patient, faceted by group. Only patients with expanded clones are shown. Number above bar refers to number of expanded cells.

medRxiv preprint doi: https://doi.org/10.64898/2026.10.28.26366745; this version posted October 30, 2026. The copyright holder for this preprint (which was not certified by peer review) is the author/funder, who has granted medRxiv a license to display the preprint in perpetuity.
It is made available under a CC-BY 4.0 International license .

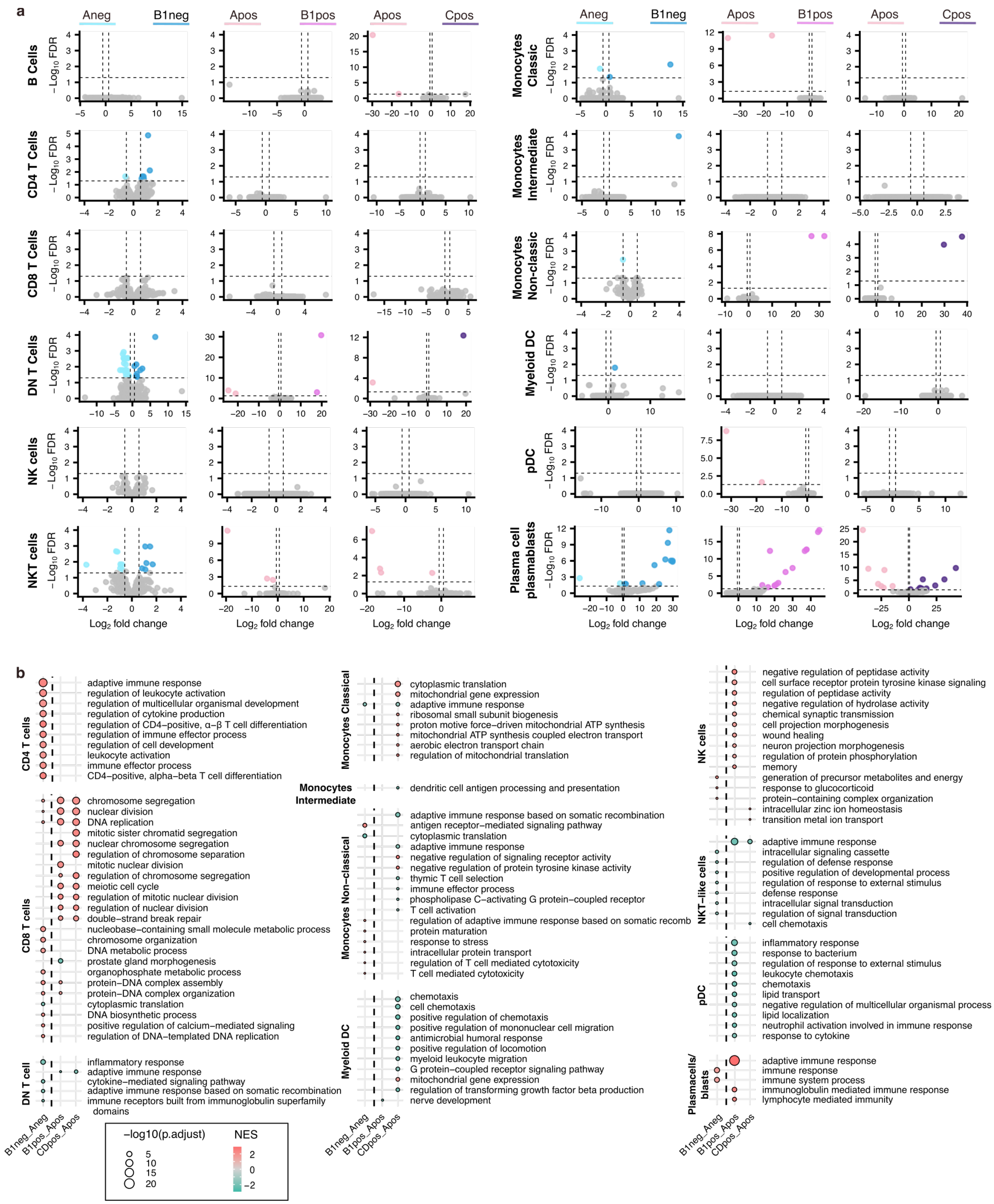

Supplemental Figure 6: Differential gene expression and gene set enrichment at the single cell level a) Volcano plot of pseudobulked differential gene expression faceted by cell type and disease severity comparisons. Each circle represents a gene: grey circles are not significant, colored circles are significant (FDR < 0.05, |Log2FC| >0.58) and colored by the disease and stage in which it is upregulated. **b)** Dot plot of the top 10 most significantly enriched gene ontology biologic process pathways for each cell type and

medRxiv preprint doi: https://doi.org/10.64898/2026.10.28.26366745; this version posted October 30, 2026. The copyright holder for this preprint (which was not certified by peer review) is the author/funder, who has granted medRxiv a license to display the preprint in perpetuity.
It is made available under a CC-BY 4.0 International license .

disease severity comparison. Circle size indicates significance and color indicates the normalized enrichment score with orange indicating activation and teal indicating suppression.

medRxiv preprint doi: https://doi.org/10.64898/2026.10.28.26366745; this version posted October 30, 2026. The copyright holder for this preprint (which was not certified by peer review) is the author/funder, who has granted medRxiv a license to display the preprint in perpetuity.
It is made available under a CC-BY 4.0 International license .

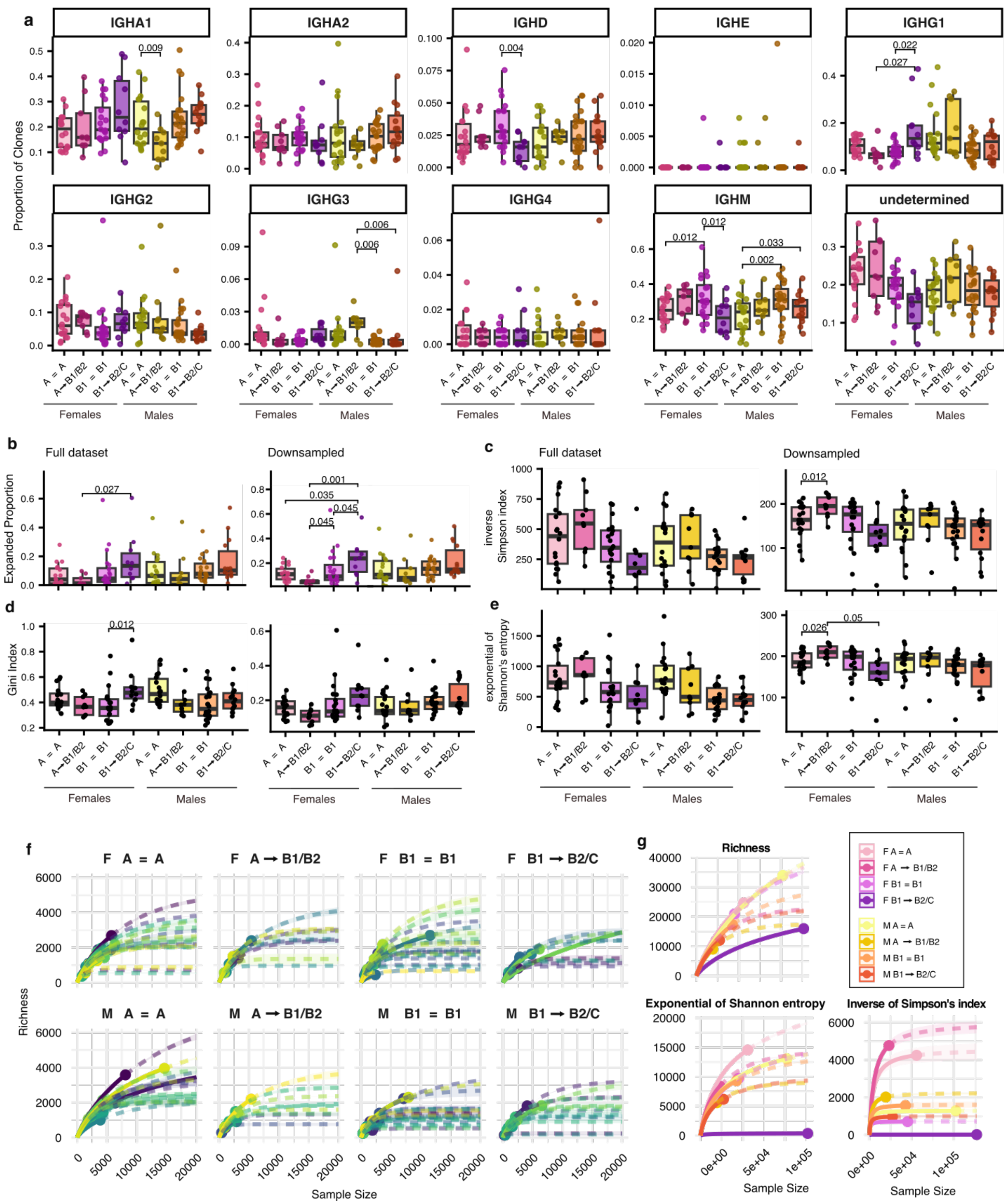

Supplementary Figure 7: Prior to advanced CCC progression in females, B cells are more class switched and less diverse. a) Isotype class proportions of downsampled clones by sex and disease stage. Statistics by beta regression adjusting for sequencing batch **b)** proportion of expanded clonotypes (> 1% of

medRxiv preprint doi: https://doi.org/10.64898/2026.10.28.26366745; this version posted October 30, 2026. The copyright holder for this preprint (which was not certified by peer review) is the author/funder, who has granted medRxiv a license to display the preprint in perpetuity.
It is made available under a CC-BY 4.0 International license .

repertoire) by sex and disease stage using the full dataset (left) or the downsampled dataset (right)**.** Statistics by beta regression. **c)** Inverse Simpson diversity, **d)** Gini coefficient, and **e)** exponential of Shannon entropy by sex and disease stage using the full dataset (left) or the downsampled dataset (right). Statistics by linear regression adjusting for sequencing batch. **(f-g)** Rarefaction curves (solid lines) with extrapolation (dashed lines) of **f)** clonotype richness vs number of contigs for each sample faceted by sex and disease stage or **g)** clonotype richness (top), exponential of Shannon entropy (bottom left), or inverse Simpson index (bottom right) vs number of contigs for each group overall. All statistical pairwise comparisons were performed using estimated marginal means with FDR correction.

medRxiv preprint doi: https://doi.org/10.64898/2026.10.28.26366745; this version posted October 30, 2026. The copyright holder for this preprint (which was not certified by peer review) is the author/funder, who has granted medRxiv a license to display the preprint in perpetuity.
It is made available under a CC-BY 4.0 International license .

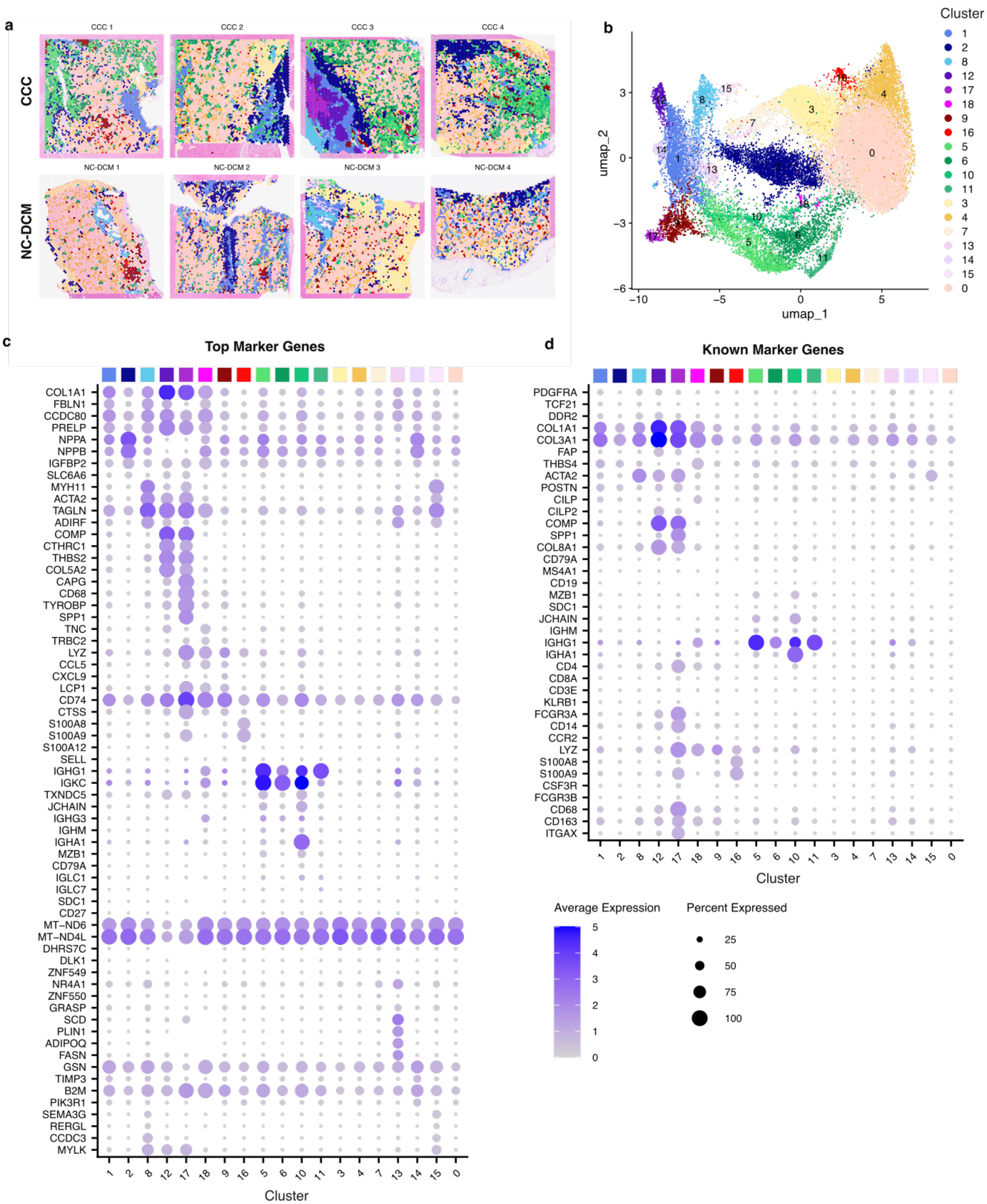

Supplemental Figure 8: Unsupervised clustering identifies various immune, fibrotic and damage-associated regions. a) Spatial plot of gene expression clusters by tissue. **b)** UMAP of gene expression clusters **c)** Top 4 marker genes for each cluster. Dot size indicates percent of spots in the cluster expressing that gene, dot color indicates average expression for that cluster. **d)** Known fibrosis and immune markers by cluster.

medRxiv preprint doi: https://doi.org/10.64898/2026.10.28.26366745; this version posted October 30, 2026. The copyright holder for this preprint (which was not certified by peer review) is the author/funder, who has granted medRxiv a license to display the preprint in perpetuity.
It is made available under a CC-BY 4.0 International license .

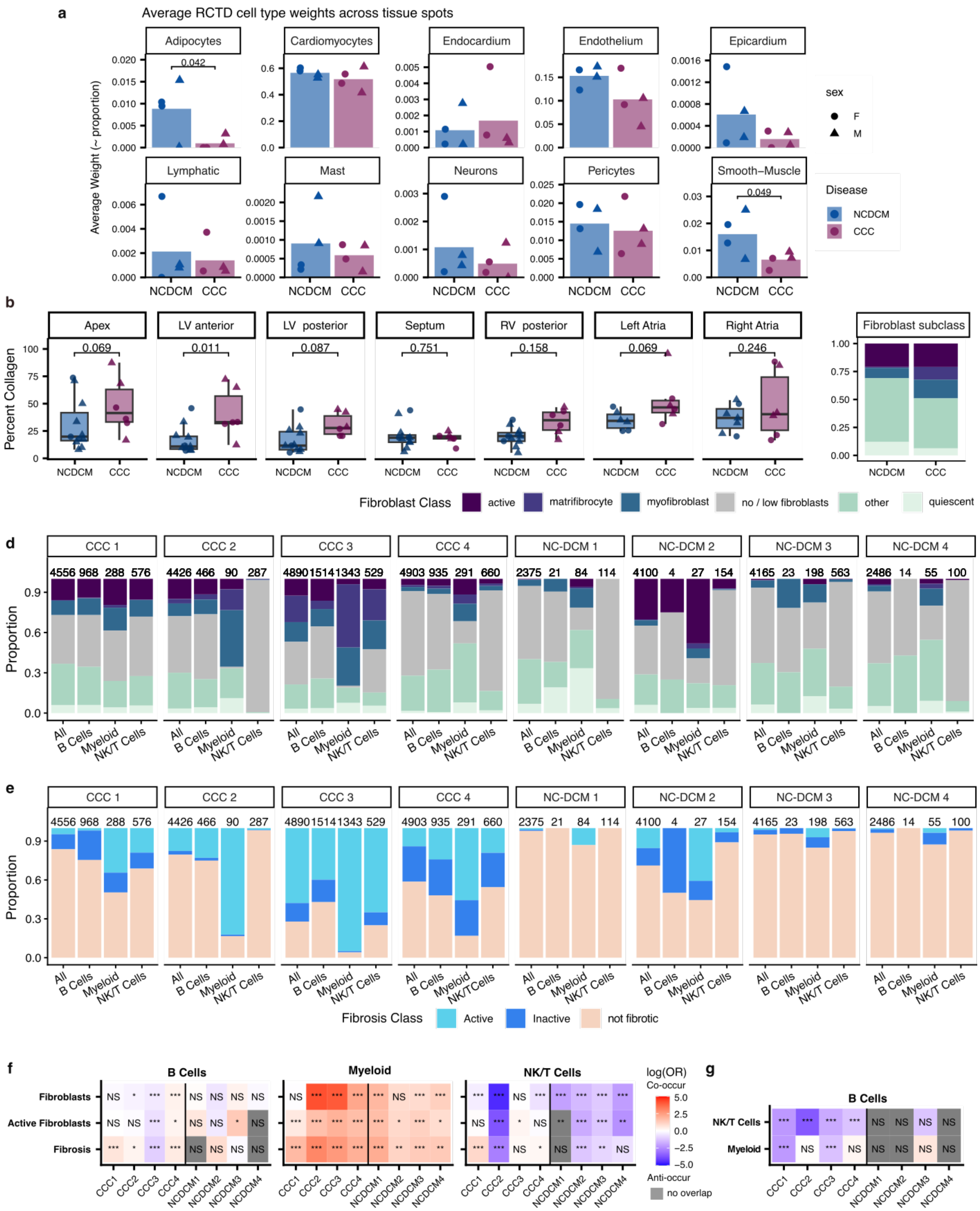

Supplemental Figure 9: Distinct immune cells are spatially associated with fibrotic niches. a) Average RCTD deconvolution weights by patient, faceted by cell type with bar indicating mean. Statistics by beta regression followed by estimated marginal means for pairwise comparisons with FDR correction. **b)**

medRxiv preprint doi: https://doi.org/10.64898/2026.10.28.26366745; this version posted October 30, 2026. The copyright holder for this preprint (which was not certified by peer review) is the author/funder, who has granted medRxiv a license to display the preprint in perpetuity.
It is made available under a CC-BY 4.0 International license .

Percentage collagen staining by tissue region and disease. Statistics by mixed-effects beta regression with fixed effects of disease * tissue region, and patient as a random effect. Estimated marginal means were used for pairwise comparisons with FDR corrections. **c)** Average fibroblast subclass proportion by disease group for spots with more than 10% fibroblasts by RCTD deconvolution. Active fibroblasts (POSTN+, CILP+, THBS4+, COMP-), myofibroblasts(ACTA2+, TAGLN+, CTHRC1+, MYH11-, COMP-), or matrifibrocytes (CILP+,CILP2+,COMP+,SPP1+,COL8A1+) **d)** Fibroblast subclass proportion for all spots, B cell-containing spots, Myeloid-containing spots, and NK/T cell-containing spots by patient. Number above bar indicates number of cells. **e)** Manual fibrosis annotation proportion for all spots, B cell-containing spots, myeloid-containing spots, and NK/T cell-containing spots by patient. Number above bar indicates number of cells. **f-g)** Heatmap of maximum likelihood estimate of log-odds ratio for co-occurrence between the respective cell types, or between cells and fibrosis, within a Visium spot for a given patient. NS - non significant. * FDR<0.05, ** FDR<0.01, *** FDR<0.001. f) co-occurrence between immune cells and fibroblasts or fibrosis. g) co-occurrence between immune cells

medRxiv preprint doi: https://doi.org/10.64898/2026.10.28.26366745; this version posted October 30, 2026. The copyright holder for this preprint
(which was not certified by peer review) is the author/funder, who has granted medRxiv a license to display the preprint in perpetuity.
It is made available under a CC-BY 4.0 International license .

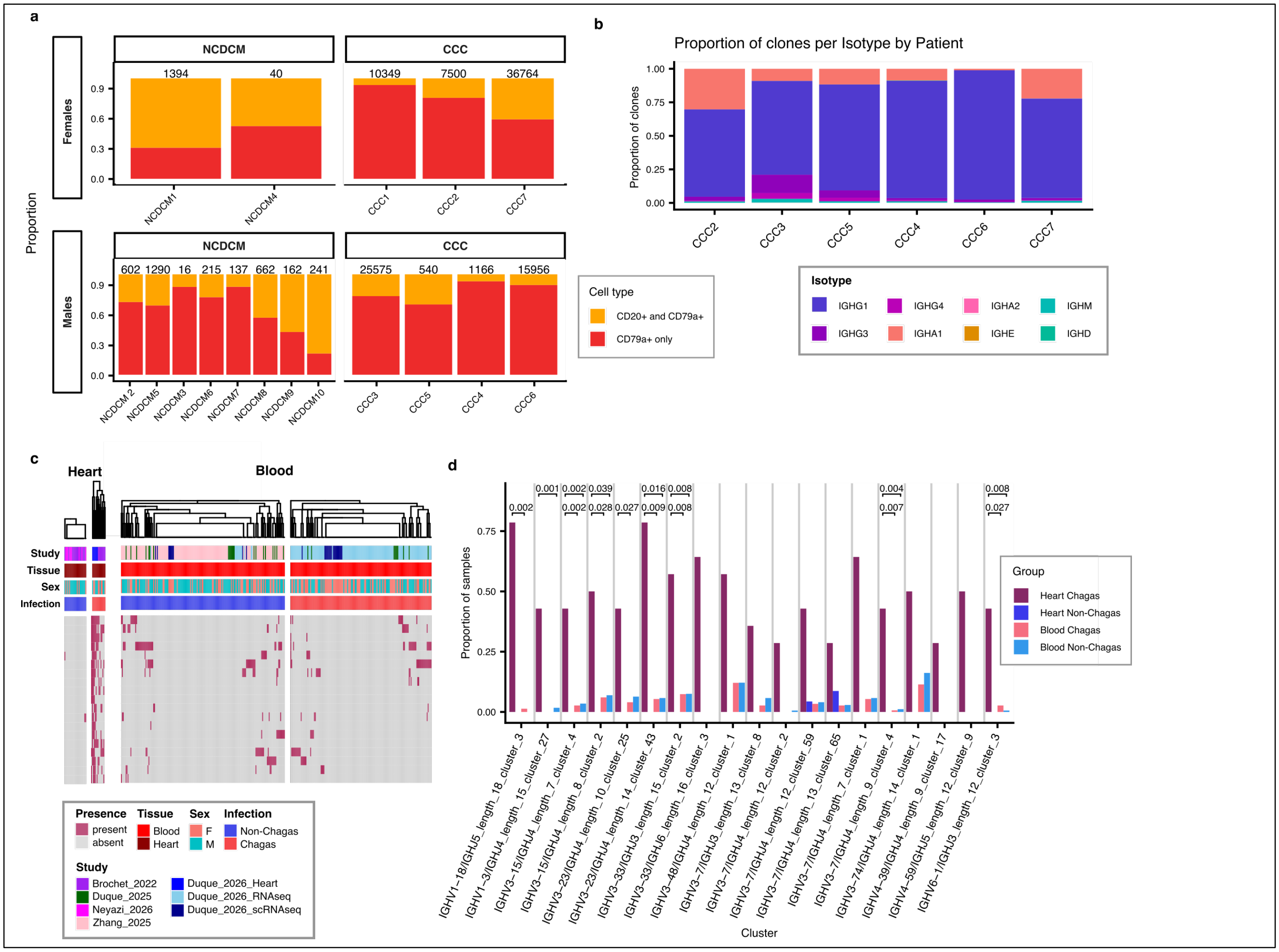

Supplemental Figure 10: CCC B cells are differentiated and have unique clonotypes. a) Stacked bar plot of the proportion of B cells identified as CD79a+ or CD20+CD79a+ by QuPath. The number above the bar indicates the absolute number of B cells identified for that patient. **b)** stacked bar plot of proportion of BCR isotypes per patient by BCRsequencing of our CCC hearts. **c)** heatmap of clonotypes (rows) detected in >50% of CCC hearts and their presence or absence in each patient (columns) from various datasets. Samples are faceted by tissue type and infection, and within each facet undergo hierarchical clustering by Euclidean distance. **d)** proportion of samples by tissue type and infection status with the clonotypes shared by >50% of our CCC hearts. Statistics by logistic regression for each cluster, adjusting for B cell sampling depth.
